# Ensemble machine learning with Bayesian optimization predicts bioactive extraction from fermented watermelon rind using natural deep eutectic solvents

**DOI:** 10.1038/s41598-026-54805-5

**Published:** 2026-05-25

**Authors:** Mostafa Khajeh, Mansour Ghaffari-Moghaddam, Afsaneh Barkhordar, Mousa Bohlooli, Didem Saloglu

**Affiliations:** 1 Department of Chemistry, Faculty of Science, University of Zabol, Zabol, Iran; 2 Advanced Materials & Manufacturing Laboratory, Central Laboratory, University of Zabol, Zabol, Iran; 3 Department of Cell & Molecular Sciences, Kharazmi University, Tehran, Iran; 4 Department of Disaster and Emergency Management, Disaster Management Institute, Istanbul Technical University, Istanbul, 34469 Turkey

**Keywords:** Microwave-assisted extraction, Natural deep eutectic solvents, Watermelon rind, Bayesian optimization, Ensemble machine learning, Bioactive compounds, Biochemistry, Biotechnology, Computational biology and bioinformatics

## Abstract

A new integrated extraction strategy was established by combining natural solvent systems (NADES), microwave-assisted extraction, and machine learning techniques for optimizing the extraction of bioactive compounds from fermented watermelon rind. The results showed that solid-state fermentation significantly improved extraction rates, compared to non-fermented controls. The fermentation pretreatment comparison was conducted under identical extraction conditions, with non-fermented watermelon rind serving as the internal control. Machine learning models optimized using Bayesian optimization were developed to describe and predict three important response variables: TPC, TFC, and antioxidant activity, as functions of four extraction variables (microwave power, temperature, time, and solid-liquid ratio). The ensemble models showed outstanding predictive capabilities with test R² values of 0.9147, 0.9088, and 0.9252 for TFC, TPC, and DPPH activity, respectively, with very low overfitting (ΔR² < 0.06). Importance analysis of the features showed temperature as the most important parameter in the extraction of bioactive compounds (importance: 0.842–0.885), followed by solid-liquid ratio. Simultaneous optimization using the validated models showed the optimal extraction conditions to be 62.5 °C, 27.7 min, 300 W, and 30 mg/mL, predicting values of 1.656 mg CE/g (TFC), 19.80 mg GAE/g (TPC), and 71.27% (DPPH activity). Solid-state fermentation enhanced extraction yields, increasing total phenolics (16.9 to 19.1 mg GAE/g), flavonoids (0.61 to 0.74 mg CE/g), and DPPH activity (66% to 75%). The developed ensemble models showed high accuracy (R² = 0.91–0.93; RMSE = 0.0812 mg/g, 0.51 mg/g, and 0.74%). Experimental validation results have confirmed the high accuracy of the model with relative errors of less than 3% for all responses. The combination of fermentation pretreatment, green extraction, and machine learning indicates promise for sustainable valorization of agricultural waste resources as sources of bioactive compounds for nutraceutical applications.

## Introduction

 Phenolic compounds are recognized as potent natural antioxidants capable of scavenging free radicals and mitigating oxidative stress, making them valuable targets for extraction from agro-industrial byproducts^1^. Watermelon (Citrullus lanatus) is a member of the family Cucurbitaceae grown extensively throughout the world; thus, it generates a large amount of agro-industrial waste. Though the edible part accounts for about 55% of the total fruit, the remaining 45%, including the skin, rind, and seeds, is usually discarded. Watermelon rind alone accounts for almost one-third of the total fruit weight and is usually underutilized, despite being edible, resulting in biomass waste and environmental issues.

The solid-state fermentation (SSF) process has been identified as a potential biological pretreatment method that is economical for improving the recovery of phenolic compounds through the enzymatic breakdown of phenolic compounds bound within the plant cell wall. In the solid-state fermentation process, microbial enzymes such as β-glucosidase and esterase degrade the ester-bound and glucosylated forms of phenolic compounds, hence increasing the availability of free phenolics for subsequent extraction. In previous research carried out on pomegranate rind and grape pomace among other agro-waste materials, it was found that solid-state fermentation increases the amount of phenolics by 15–40% as opposed to the control sample without fermentation^2,3^. While there has been increasing research on watermelon rind as a bioactive source, the utilization of solid-state fermentation as a pre-treatment technique for watermelon rind has not received much attention. The fermentation-assisted extraction technique has been applied to different matrices of agricultural waste, such as pomegranate rind and grape pomace, with marked increases in phenolic content observed^2,3^. The integration of solid-state fermentation pre-treatment with green extraction methods such as NADES-mediated MAE is an innovative approach yet to be exploited for watermelon rind bioactives.

Recently, there has been emphasis on the utilization of watermelon rind in sustainable food systems and circular bioeconomy approaches^4–6^. Watermelon rind has emerged as a potential source of bioactive phytochemicals, especially phenolic and flavonoid compounds, which are mainly responsible for its antioxidant properties. These compounds have been found to be involved in the scavenging of reactive oxygen species and have been linked to various biological activities, such as antihypertensive, antidiabetic, anticancer, and cardioprotective properties. Various efficient extraction techniques could increase phenolic and flavonoid losses while preserving their antioxidant potential. Research suggests that there is a strong positive correlation between total phenolic and total flavonoid content with regard to their antioxidant activities; therefore, the rind of watermelon is a good source of antioxidants that can be used in nutraceuticals and pharmaceuticals^7,8^. Traditional techniques like maceration, heat-assisted extraction, and Soxhlet extraction have already been used for the isolation of phenolic compounds and flavonoids from the watermelon rind; however, these techniques suffer from poor efficiency, long extraction time, and the possible degradation of heat-sensitive active molecules^9,10^.

Conventional techniques have been extensively utilized for isolating natural compounds from plants such as the watermelon rind. Examples of some of the conventional techniques that have been employed for isolating various phytochemicals from plant materials include maceration, Soxhlet extraction, heat-assisted extraction, and enzyme-assisted extraction^9,10^. However, some of the limitations associated with these techniques include low yields, extended extraction times, and the utilization of high temperatures that may lead to the destruction of heat-labile bioactive compounds^10^. For this reason, there has been an increased trend toward utilizing modern extraction methods, which include microwave-assisted extraction, pulsed electric field-assisted extraction, accelerated solvent extraction, ultrasound-assisted extraction, and supercritical fluid extraction. All of these methods are effective in producing high-yield isolates of phenolic compounds from various sources of foods and food byproducts^11–15^.

In recent years, natural deep eutectic solvents (NADES), also known as green solvents, have gained increasing popularity in replacing traditional organic solvents because of their renewability, non-toxicity, low cost, and versatility in functionality^16^. NADES are normally made up of natural compounds such as sugars, quaternary ammonium salts, amino alcohols, and carboxylic acids. These compounds are rich in hydroxyl or carboxyl groups and have been shown to form extensive hydrogen bonding interactions with target compounds, thus increasing both the yield and stability of the extraction^17^. Compared to traditional organic solvents, NADES have several advantages, including being environmentally friendly, biodegradable, biocompatible, and non-toxic. They are effective in the extraction of a variety of bioactive compounds such as polyphenols, flavonoids, and polysaccharides^18^. To reduce the time needed for extracting compounds from various plant sources, researchers have begun utilizing microwave-assisted extraction (MAE) as a component of their extraction methodology; specifically, by applying the use of NADES to extract compounds in conjunction with MAE. Microwave energy is utilized to create and enhance interactions between the extraction solvent and the bioactive compound(s) target, increasing extraction rates significantly. Consequently, the incorporation of NADES and MAE will allow for the maintenance of the environmental advantages of NADES while improving the efficiency and selectivity of extraction methods. Therefore, the combination of NADES and MAE is an emerging sustainable method for the extraction of bioactive compounds from plant materials. NADES have great potential to extract bioactive compounds of plant origin due to their environmental friendliness, preparation ease, and their biodegradability^19^.

However, traditional experimental optimization techniques, although successful, can be time- and resource-consuming, thereby requiring the use of sophisticated computational models. Machine learning (ML), especially ensemble learning, has been a game-changer in the field of food bioactive compound studies, providing far superior capabilities in modeling complex nonlinear relationships and optimizing extraction conditions^20,21^. Bayesian optimization has revealed promising results in bioprocess engineering, with numerous studies reporting a substantial reduction in experimental burden compared to traditional design of experiments, while achieving comparable or superior optimization outcomes^21^. Additionally, ensemble learning models, which combine base learners, have been shown to be highly effective at predicting the bioactive properties of food compounds^20,22^. The combination of Bayesian optimization and ensemble ML models is a state-of-the-art computational tool that has the potential to greatly accelerate the discovery and quantification of bioactive compounds from food waste materials, thereby playing an important role in the development of circular economy strategies.

Therefore, the purpose of this research was to investigate the combined effect of the solid-state fermentation pretreatment process and the NADES-based microwave-assisted extraction method for the extraction of bioactive compounds from watermelon rind. In particular, the research was set out to: (i) compare watermelon rinds before and after fermentation in terms of their total phenolic contents, total flavonoid contents, and antioxidant capacities; (ii) assess the impact of microwave power, temperature, extraction time, and solid-to-liquid ratio on the extraction efficiency; (iii) generate machine learning models that were optimized through Bayesian optimization techniques for the prediction of TPC, TFC, and DPPH responses; and (iv) establish the optimum extraction conditions for achieving maximum responses.

## Experimental

The whole experimental procedure included three successive steps: (1) watermelon rind was dried, ground, and allowed to undergo yeast fermentation process for better cellular membrane disruption and active ingredient liberation; (2) ternary NADES (citric acid: urea: sucrose) was independently prepared via ultrasound homogenization to achieve full eutectic point formation; and (3) fermentation products were subjected to extraction with NADES solvent through the application of microwave-assisted extraction at different powers, temperatures, times, and solid-to-liquid ratios depending on the designed experiment (Table 1). Analytical determinations (total phenolic content, total flavonoid content, and free radical scavenging activity) were conducted on microwave extracts.

### Materials

Chemicals and reagents utilized in this research were either bought from Merck (Darmstadt, Germany) or otherwise noted as analytical grade. The watermelon rind was dried and authenticated before the extraction experiment. The analytical standard reagents used include Folin-Ciocalteu reagent, aluminum chloride, gallic acid, catechin, and 2,2-diphenyl-1-picrylhydrazyl (DPPH) radical. All aqueous solutions were prepared using deionized water purchased from a Milli-Q Water Purification System (Millipore, Bedford, MA, USA).

### Watermelon rind preparation

Watermelons were bought from the local market in Zabol, Iran. The watermelon’s surface was then washed with distilled water to eliminate any dust and dirt. The watermelons were then peeled manually to separate the outer skin from the inner flesh. The flesh was then sliced into equal pieces, 2–3 mm thick, to enable efficient drying. The watermelon slices were then dried in a shaded area to avoid the degradation of heat-sensitive bioactive compounds. Drying was continued until the weight of the watermelon slices became constant, with less than 0.5% variation in weight. At this point, the water content in the watermelon slices was assumed to be negligible. The dried rind of watermelon was ground to a fine powder using a laboratory mill. The powder was then sieved using a 40-mesh sieve to ensure uniformity in particle size. The sieved powder was stored in a tightly sealed container at 4 °C in the dark to preserve its stability and prevent oxidative degradation until the chemical and functional analyses. The solid-state fermentation process was done as described by D’Agostino et al.^2^. Dry yeast (29.17 mg/mL) was mixed with distilled water (17 mL) at room temperature for 10 min. Watermelon rind powder (20.0 g) was then added and mixed for 20 min. Following this process, after pouring the mixture into sterile glass vials covered with aluminum foil and incubating at 30 °C for 48 h, the fermented watermelon powder was air-dried at room temperature until a fixed weight was reached. The dried and ground rind was chosen as the fermentation material instead of the fresh rind to ensure constant moisture levels and avoid variation due to intrinsic microorganisms that may be present. This method conforms to SSF methods used for agro-industrial residues; wherein precise control of water activity is essential for maximum enzyme efficiency and avoidance of microbial contamination.

### NADES procedure

A ternary natural deep eutectic solvent (NADES) was formulated by mixing citric acid, urea, and sucrose (each 1.0 mol/L) in deionized water. Citric acid, urea, and sucrose were initially added to a glass vessel, followed by the slow addition of distilled water with continuous magnetic stirring until a uniform mixture was formed. The mixture was then treated with ultrasonic waves for 15 min at room temperature to enhance molecular interactions and the formation of hydrogen bonding networks characteristic of NADES. The mixture was then gently heated to 50 °C on a hot plate with continuous stirring for 25 min to confirm the whole integration of the components and the formation of eutectics. A second ultrasonic treatment was performed for 10 min to ensure a uniform dispersion and stability of the solvent system. The formulated NADES was allowed to cool to 4 °C and stored in a sealed amber glass container until analysis. The solvent formulation was modified from Khajeh et al.^23^ and adapted to the requirements of the current study. All three constituents of the NADES, citric acid, urea, and sucrose, are naturally available, readily degradable, and have low ecotoxicity. Citric acid is found in citrus fruits, sucrose is a plant-based sugar, and urea is a natural biological product. The synergistic interactions of all three molecules create an extensive hydrogen bonding network to substitute volatile organic solvents without affecting the efficiency of phenolic/flavonoids compound extraction, in accordance with the principles of green chemistry. In the case of ultrasonic treatment of the NADES before its application, this treatment method was strictly utilized only as a homogenizing technique to facilitate the creation of eutectic bonds between NADES constituents. Since no plant material was present during the ultrasonic treatment, this step was employed solely for solvent preparation and cannot be considered ultrasonic-assisted extraction of bioactive compounds from watermelon rind. The NADES composition was fixed based on prior experience, literature guidance^23^, and preliminary laboratory evaluations. Hence, optimization of the NADES composition was considered beyond the scope of the present study.

### Experimental design and process parameters

In this study, a full-scale experimental design determined the effect of the joint extraction and analysis process of bioactive compounds found in watermelon rind using microwave-assisted extraction (MAE) on the yield of various parameters Khajeh et al.^23^. The overall goal of the study was to optimize the MAE process to maximize the yields of three major parameters, which include TFC, TPC, and antioxidant activity, expressed as DPPH radical scavenging capacity. A total of 50 experimental trials were conducted to determine the influence of four independent process variables, as listed in Table 1, on the yield of each of these parameters. The four independent variables studied in this work were identified based on preliminary experiments and existing literature, with their ranges identified through preliminary screening:

**Table 1:** Experimental variables with actual responses in the MAE of bioactive compounds from watermelon rind.

No.	Power (W)	Temperature (°C)	Time (min)	Mass/volume (mg/mL)	Total Phenol (mg/g)	Total Flavonoids (mg/g)	DPPH%
1	100	40	5	20	19.69	0.63	72.64
2	100	40	30	40	12.55	0.89	57.86
3	100	70	5	40	12.84	1.14	65.72
4	100	70	30	20	22.65	1.75	65.44
5	500	40	5	40	10.94	0.62	64.42
6	500	40	30	20	14.98	0.89	69.16
7	500	70	5	20	15.79	1.35	69.20
8	500	70	30	40	12.97	1.72	86.18
9	300	55	17.5	30	13.44	1.01	64.35
10	300	55	17.5	30	13.96	1.12	77.56
11	300	55	17.5	30	15.77	1.37	70.87
12	100	55	17.5	30	12.29	0.97	61.07
13	500	55	17.5	30	17.87	1.05	74.19
14	300	40	17.5	30	14.24	0.93	63.00
15	300	70	17.5	30	19.64	1.65	69.33
16	300	55	5	30	14.62	0.85	63.52
17	300	55	30	30	15.54	1.25	72.43
18	300	55	17.5	20	18.74	1.16	73.31
19	300	55	17.5	40	11.59	0.86	72.62
20	200	45	10	25	14.77	0.79	70.09
21	200	45	25	35	13.42	1.01	79.20
22	200	65	10	35	17.01	1.20	68.75
23	200	65	25	25	21.54	1.75	70.56
24	400	45	10	35	13.28	0.70	70.00
25	400	45	25	25	16.14	0.91	68.71
26	400	65	10	25	19.54	1.25	73.54
27	400	65	25	35	18.53	1.58	78.58
28	150	50	20	30	16.48	1.01	64.33
29	450	60	15	30	10.72	1.34	76.72
30	250	60	25	25	18.31	1.54	77.94
31	350	50	10	35	11.55	0.66	73.39
32	250	50	15	40	14.16	0.86	72.34
33	350	60	20	20	16.14	1.37	67.09
34	150	60	25	30	20.22	1.31	67.42
35	450	50	10	30	15.54	0.81	66.03
36	250	45	20	35	10.59	1.19	68.42
37	350	65	15	25	17.71	1.57	70.43
38	150	65	20	40	10.71	1.26	72.30
39	450	45	25	20	16.41	1.04	59.53
40	200	55	10	30	12.27	0.79	65.18
41	400	55	25	30	16.87	1.16	70.00
42	300	45	10	25	15.45	0.75	69.71
43	300	65	25	35	17.07	1.60	71.89
44	250	55	20	20	17.61	1.11	72.64
45	350	55	15	40	11.61	0.93	72.26
46	200	60	20	30	16.69	1.26	68.35
47	400	50	15	30	14.34	0.88	70.38
48	150	55	17.5	30	14.92	0.98	64.09
49	450	55	17.5	30	15.55	1.01	74.65
50	300	55	17.5	30	14.27	1.04	70.95

### Microwave-assisted extraction procedure

Microwave-assisted extraction was carried out as per the experimental design given in Table 1. For each experiment, a known weight of powdered sample was mixed with the extraction solvent under the given conditions. All experiments were carried out in triplicate. The sample mixture was loaded into a microwave extraction vessel and carried out using a microwave extraction system (Sorisole, Italy) provided with temperature and power control. The extraction temperature was continuously measured using a fiber-optic temperature sensor and controlled to ± 1 °C of the set value using an automated feedback control system. After completion of the programmed extraction time, the vessel was quickly cooled to ambient temperature. Following centrifugation at a speed of 5000 rpm for a period of ten minutes, the supernatant was separated from any insoluble material by filtration through Whatman No. 1 filter paper. The filtered extracts were stored in sealed amber-colored glass containers at 4 °C and analysed within 24 h for total phenolics (TPC), total flavonoids (TFC), and antioxidant activity.

### Analytical methods

#### Total flavonoid content (TFC)

The TFC was determined by the aluminum chloride colourimetric assay that was modified according to Aryaeifar et al.^24^. In brief, an aliquot of diluted sample (10 µL) was added to a solution of sodium nitrite (5% (w/v) sodium nitrite, 100 µL), and incubated for 5 min, followed by the addition of a solution of aluminum chloride (10% aluminum chloride (w/v), 100 µL). AlCl3 and NaNO2 were allowed to react for 5 min without mixing. After 5 min had passed from the addition of AlCl3 and NaNO2, 1.0 molar of sodium hydroxide solution (1.0 mL) and distilled water to a final volume of 5.0 mL were added. The final volume produced by this reaction was vigorously mixed before allowing it to rest for 15 min. After 15 min, the absorbance was measured at 510 nm on a UV-Vis spectrophotometer against a reagent blank. The total flavonoid content was determined from a calibration curve constructed using catechin as the reference standard and expressed as mg of catechin equivalents (CE) per gram of dry weight (g CE/g).

#### Total phenolic content (TPC)

A colorimetric method was used to determine the total phenolic content (TPC) using the Folin-Ciocalteu reagent. The colorimetric method can be summarized as follows: The sample solution was diluted with distilled water until it had the appropriate level of concentration for analysis. A sample volume of 100 µL of the diluted sample solution was combined with 500 µL of the Folin-Ciocalteu reagent. Following an incubation period of 5 min, 1.5 mL of 20% (w/v) sodium carbonate solution was added to the mixture. After another incubation period (5 min) at room temperature (25 °C) in the dark, the total volume of the reaction mixture was adjusted to 10 mL via the addition of distilled water and allowed to incubate for 2 h at room temperature and in the dark. Absorbance was then measured at 765 nm using UV Vis spectroscopy. Using a standard curve based on gallic acid, the TPC value for the sample was calculated as milligrams of gallic acid equivalent per gram of dry sample^24^.

#### DPPH radical scavenging activity

The DPPH (2,2-Diphenyl-1-picrylhydrazyl) radical scavenging assay was used to assess the antioxidant activity of the compounds. The solution of DPPH is prepared by dissolving 24 mg of it in 100 mL of methanol, followed by filtering to obtain a clear DPPH solution. To conduct the assay, a 100 µL aliquot of the diluted sample solution is combined with 3.0 mL of DPPH solution, and the mixture is left to react in the dark at room temperature for 30 min (the time required for complete reaction between the antioxidants and DPPH). The control assay used the same reaction conditions, but the 100 µL sample was replaced with methanol^24^. After completing the reaction, the colored solution at 517 nm was quantified with a UV-Vis spectrophotometer, using methanol as the blank. The DPPH radical scavenging activity of the compounds was calculated according to:

1 $$DPPH{\text{ }}scavenging{\text{ }}\left( \% \right){\text{ }} = {\text{ }}\left[ {\left( {A_{{control}} - {\text{ }}A_{{sample}} } \right){\text{ }}/{\text{ }}A_{{control}} } \right]{\text{ }} \times {\text{ }}100$$

where A_control_ is the absorbance of the DPPH solution without the extract, and A_sample_ is the absorbance of the DPPH solution with the extract.

### Data processing and machine learning methodology

#### Feature engineering and data preprocessing

Because of the non-linear interaction and relationship of the variables involved in the extraction procedure, an expanded feature space was created using four primary variables, i.e., power (P, W), temperature (T, °C), time (t, min), and solid-liquid ratio (S/L, mg/mL). By employing feature engineering, new features were created using different transformations of the primary variables, which were aimed at capturing the physical and chemical phenomena involved in the extraction process. The feature engineering strategy was adapted based on a preliminary analysis of each response variable’s sensitivity and the apparent complexity of their interaction as revealed by the experimental data.

The features created were aimed at capturing meaningful physical characteristics of the extraction process, which included the following categories:

##### Two-way interaction terms to model synergistic effects among process variables

- P × t (Power-Time interaction), T × t (Temperature-Time interaction), P × T (Power-Temperature interaction), P × S/L (Power-Ratio interaction), T × S/L (Temperature-Ratio interaction), t × S/L (Time-Ratio interaction).

##### Polynomial (quadratic) terms to model non-linear curvature effects

- P², T², t², (S/L)²:

##### Ratio terms to represent relative influences and dimensionless process groups

- P/T, P/t, T/t, P/S/L:

##### Energy-related composite features

- P × t (Total energy input), (P × t)/S/L (Energy density).

The selection and implementation of these engineered features were done in a data-driven fashion, specific to each response variable. For those models where preliminary analysis revealed primarily linear or mildly non-linear behavior, a subset of the most physically relevant interaction and energy-related features was utilized to ensure an appropriate number of features to samples ratio. For response variables that demonstrated complex, highly non-linear behavior as a function of extraction conditions, a broader set of features, including polynomial and ratio-based terms, was utilized to adequately capture the intricate relationships.

#### Feature standardization

To ensure the equal contribution of all features during the training of the model and avoid scale-dependent bias in the optimization algorithms, all the features, both original and engineered, were standardized using z-score normalization:

2 $$X_{{scaled}} = {\text{ }}\left( {X{\text{ }} - {\text{ }}\mu } \right){\text{ }}/{\text{ }}\sigma$$

where X is the original feature value, µ is the mean, and σ is the standard deviation. Importantly, the parameters (µ and σ) were calculated only from the training set and then applied to the test set to avoid data leakage and biased model assessment. This method of normalization ensures that all features are centered at 0 and have a standard deviation of 1, so that features that are inherently larger in scale, such as power in W and temperature in °C, contribute equally to model training. All the standardized features were then fed into the various machine learning algorithms. For some models that used a stacking ensemble architecture, both the input features and the target variables were standardized to improve the numerical stability of the meta-learning algorithms. The predictions were then inverse-transformed to the original scale for easier interpretation and reporting.

#### Bayesian optimization framework

Bayesian optimization (BO) was used as a systematic and efficient optimization strategy for machine learning model hyperparameter tuning. Bayesian optimization is different from traditional methods like “grid” and “random” search in that it uses a statistical model for the objective function, building up some prior information on the objective function over time, and then it uses this information to direct its search through the space of hyperparameters, concentrating on promising parts of the search space. The optimization problem can be described as:

3 $$\theta *{\text{ }} = {\text{ }}\arg {\text{ }}\min _{{\theta \in \Theta }} f\left( \theta \right)$$

where θ* shows the optimal hyperparameter configuration, Θ represents the hyperparameter search space, and f(θ) shows the objective function defined as the negative cross-validated R² score:

4 $$f\left( \theta \right){\text{ }} = {\text{ }} - 1/K{\text{ }}\Sigma _{{k = 1}} ^{K} R_{k} \left( \theta \right)$$

where K = 5 is the number of cross-validation folds, and R²_k_(θ) represents the coefficient of determination for the k-th fold by hyperparameters θ.

A Gaussian process (GP) was employed as a probabilistic surrogate model for the objective function:

5 $$f\left( \theta \right) \sim GP(m(\theta ),{\text{ }}k(\theta ,\theta \prime ))$$

where m(θ) is the mean function (assumed to be zero) and k(θ, θ′) is the Matern covariance kernel with ν = 2.5:

6 $$k(r) = (1 + \sqrt {(5r/l + 5r^{2} /3l^{2} )} )\exp ( - (5r/l))$$

where r = ||θ − θ′|| is the distance between two hyperparameter configurations, and l is the length-scale parameter. This kernel provides a reasonable compromise between continuity and flexibility.

The acquisition function used to identify the next hyperparameter combination to evaluate on this surface is based on Expected Improvement (EI).

7 $$EI\left( \theta \right){\text{ }} = {\text{ }}\left( {f\left( {\theta \_best} \right){\text{ }} - {\text{ }}\mu \left( \theta \right)} \right)\Phi \left( Z \right){\text{ }} + {\text{ }}\sigma \left( \theta \right)\varphi \left( Z \right)$$

where Z = (f(θ_best) − µ(θ))/σ(θ), f(θ_best_) represents the current best observed function value, Φ = Cumulative Distribution Function, and φ = Probability Density Function. The acquisition function automatically balances the exploitation of known good areas with the exploration of uncertain areas. For the flavonoid, radical scavenger, and phenolic content models, CatBoost gradient boosting regressors were optimized. Table 2 shows the hyperparameter search space for this study.

**Table 2:** Hyperparameter ranges used for Bayesian optimization of CatBoost models predicting total flavonoid, radical scavenger, and phenolic contents, including iterations (niter), learning rate (η), tree depth (dmax), L2 regularization (λ), and subsampling ratio (ρ).

Hyperparameter	Search Range	Description
n_iter_ (iterations)	50–150	Number of boosting rounds
η (learning rate)	0.02–0.15	Step size shrinkage factor
d_max_ (tree depth)	3–5	Maximum depth of decision trees
λ (L2 regularization)	3.0–12.0	L2 leaf regularization coefficient
ρ (subsample ratio)	0.7–0.95	Random row sampling fraction

In addition, Bayesian optimization was performed for a variety of base learners, including Gradient Boosting Regressor, Random Forest, Support Vector Regression, and Multi-Layer Perceptron, for which hyperparameter spaces are tailored to each model architecture. The optimization process for each model architecture involves 30 to 50 sequential iterations, each of which takes approximately 2 to 5 min of computation on a standard workstation.

#### Ensemble learning architecture

To enhance the robustness of the prediction results and reduce the likelihood of overfitting to particular model configurations, various ensemble learning techniques were adopted. For the model developed to predict the total flavonoid content, an ensemble model based on weighted voting was created with the following three different regression models:

1. CatBoost Gradient Boosting: With optimal hyperparameters tuned via Bayesian optimization to provide the primary high-performance model with the ability to naturally handle categorical features and ordered boosting to combat overfitting.
2. Random Forest: With 100 trees, maximum depth of 5, minimum samples per split of 5, and minimum samples per leaf of 3 to provide variance reduction via bootstrap aggregation.
3. Ridge Regression: A linear regression model with an L2 penalty coefficient α set to 5.0 to provide the ability to capture linear relationships and provide stable extrapolation properties.

The weighted average prediction was made based on the predictions made by the three different models:

8 $$\hat{y}_{{ensemble}} = {\text{ }}\Sigma _{{i = 1}} ^{M} w_{i} \hat{y}_{i}$$

where M = 3 shows the number of base learners, w_i_ is the weight assigned to the i-th model (with Σw_i_ = 1), and ŷ_i_ is the prediction from the i-th model. The weights were set to w = [0.6, 0.2, 0.2] for CatBoost, Random Forest, and Ridge regression, respectively, based on their individual cross-validation performance.

For the total phenolic content and DPPH antioxidant activity models, stacking ensemble architectures were used, wherein predictions made by several optimized base learners (RF, GBR, SVR, MLP) were used as inputs for a meta-learner (Ridge regression), which was trained using out-of-fold predictions.

#### Model validation and performance metrics 

The entire dataset of experiments, which had 50 observations, was divided into the training set and the separate test set, with 80% and 20%, respectively, by stratified random sampling. The training set was only used for hyperparameter optimization, feature engineering, and ensemble model building, while the test set was only used for the evaluation of the models.

The performance of the models was evaluated based on three regression metrics:

##### Coefficient of determination (R²): measuring the proportion of variance in the response variable explained by the model

9 $$\:{R}^{2}=1-\frac{S{S}_{res}}{S{S}_{tot}}=1-\frac{\sum\:_{i=1}^{n}({y}_{i}-{\widehat{y}}_{i}{)}^{2}}{\sum\:_{i=1}^{n}({y}_{i}-\stackrel{\prime }{y}{)}^{2}}$$

##### Root mean squared error (RMSE): quantifying average prediction error in the original units

10 $$\:RMSE=\sqrt{\frac{1}{n}\sum\:_{i=1}^{n}({y}_{i}-{\widehat{y}}_{i}{)}^{2}}$$

##### Mean absolute error: providing a robust, interpretable measure of typical prediction deviation

11 $$\:\mathrm{M}\mathrm{e}\mathrm{a}\mathrm{n}\:\mathrm{a}\mathrm{b}\mathrm{s}\mathrm{o}\mathrm{l}\mathrm{u}\mathrm{t}\mathrm{e}\:\mathrm{e}\mathrm{r}\mathrm{r}\mathrm{o}\mathrm{r}=\frac{1}{n}\sum\:_{i=1}^{n}\mid\:{y}_{i}-{\widehat{y}}_{i}\mid\:$$

where n is the number of samples, y_i_ is the observed value, ŷ_i_ is the predicted value, and ȳ is the mean of observed values.

For evaluating potential generalization ability, and identifying overfitting, we conducted a stratified five-fold cross-validation for hyperparameter tuning on the training dataset. Mean ± SD of cross-validation results were reported an average across folds; overfitting level calculated from differences between correlation coefficients of training and test datasets using the equation below:

with ΔR² < 0.05 considered excellent (minimal overfitting), 0.05 ≤ ΔR² < 0.10 acceptable, and ΔR² ≥ 0.10 indicating potential overtraining requiring model revision.

## Results and discussion

### Effect of fermentation on bioactive compound extraction from watermelon rind

**Fig. 1:**
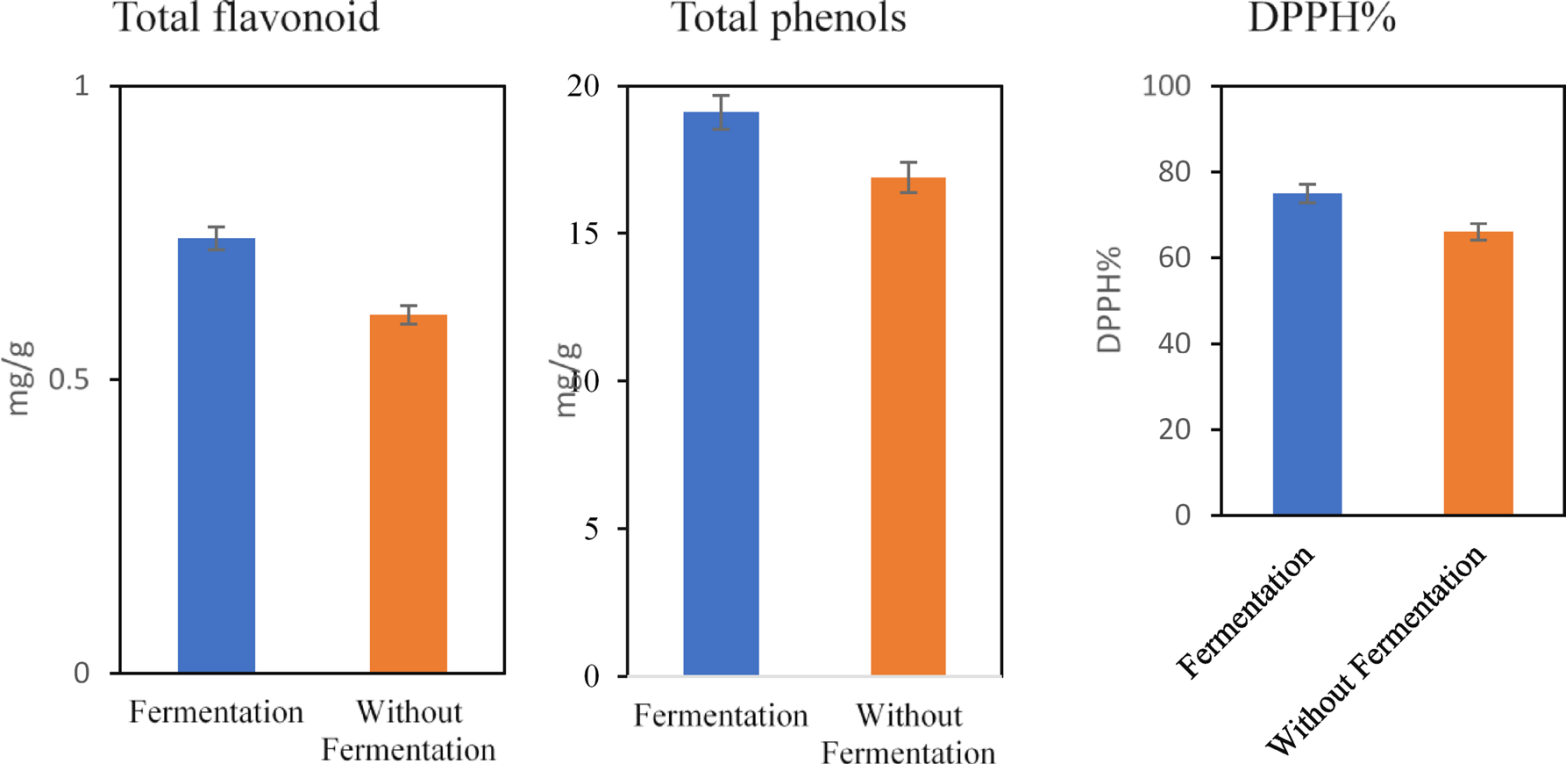
Comparison of TFC, TPC, and antioxidant activity (DPPH%) extracted from watermelon rind using deep eutectic solvent–based microwave-assisted extraction with and without fermentation.

In this study, we examined how the fermentation of powdered watermelon rind affected the amount of bioactive compounds and antioxidant activity within the powder after extraction via microwave-assisted extraction (MAE)-NADES. We performed this extraction at 50 °C and 100 watts of power for 20 min from an initial concentration of 20 mg/mL of powder (a source of solid for extraction) to determine how these different extraction conditions impacted the total quantity of bioactive compounds extracted from the sample through the process of MAE. The overall results from the study demonstrated that, as compared to non-fermented powders of watermelon rind, fermented powders of watermelon rind contain greater amounts of bioactive compounds than those powders that were not subjected to fermentation. As shown in Fig. 1, the fermentation process showed a significant improvement in the amount of total phenolics, total flavonoids, and antioxidant activities extracted from the watermelon rind by the NADES-based microwave-assisted method. Fermented watermelon rinds have been found to contain more phenolic and/or flavonoid compounds than control samples due in part to the degradation of structural polysaccharides found in the cell walls (cellulose, hemicellulose, pectin) through the action of enzymes and/or microorganisms, leading to a greater release of phenolic compounds from the cell walls. During fermentation, the presence of β-glucosidase and esterase (both produced during fermentation) facilitates the breakdown of phenolic conjugates, resulting in free phenolic compounds found in the resulting extracts^3,25^. In addition, according to Fig. 1, the antioxidant activity of the watermelon rind extracts, measured by DPPH free radical scavenging activity, was higher in the fermented extracts than in the control, which is likely attributed to the increased quantity of phenolic compounds extracted from the watermelon rind during fermentation. Therefore, the increased levels of phenolic compounds in the fermented extracts may have also contributed to their enhanced antioxidant potential^3^. The fermentation of watermelon rind yielded superior levels of phenolic compounds; therefore, it was selected for further optimization studies. The comparative study was purposely set up to identify the effect of fermentation pre-treatment since both fermented and non-fermented watermelon rind powder extracts were prepared using the same conditions of temperature (50 °C), power (100 W), time (20 min), and concentration (20 mg/ml). All optimization studies described in Sect. 3.2–3.4 were carried out using only fermented samples, and therefore any variation observed is a result of extraction optimization rather than fermentation. Fermentation was done at 30 °C for 48 h, which is an ideal temperature that facilitates optimal metabolic activities in the yeast without the risk of degradation of the phenols through heat exposure. By covering the fermentation vessels with foils, we created an aerobic atmosphere, preventing the production of ethanol that would have accelerated the oxidative destruction of polyphenols. During the fermentation process, enzymes like β-glucosidases and esterases released by the yeast act on the phenolic conjugates, hydrolyzing them to produce free phenols that possess potent antioxidant activities^3,25^. Drying of the fermentation products was carried out aerobically under ambient air conditions, since the use of higher temperatures for the same purpose would result in loss of phenolic content as thermally labile flavonoids would be easily destroyed^2^. The moderate fermentation temperature (30 °C) also maintained yeast viability throughout the 48 h incubation, ensuring sustained enzymatic activity for maximum cell wall depolymerization and phenolic release.

### Machine learning model performance

The developed machine learning models exhibited excellent prediction performance for all three response variables, and the ensemble-based architectures successfully modeled the intricate non-linear interactions between microwave-assisted extraction conditions and bioactive compound yields. Furthermore, the combination of Bayesian hyperparameter optimization and ensemble-based machine learning strategies achieved high accuracy, low overfitting, and excellent generalization performance.

#### Total flavonoid content (TFC) prediction

**Fig. 2:**
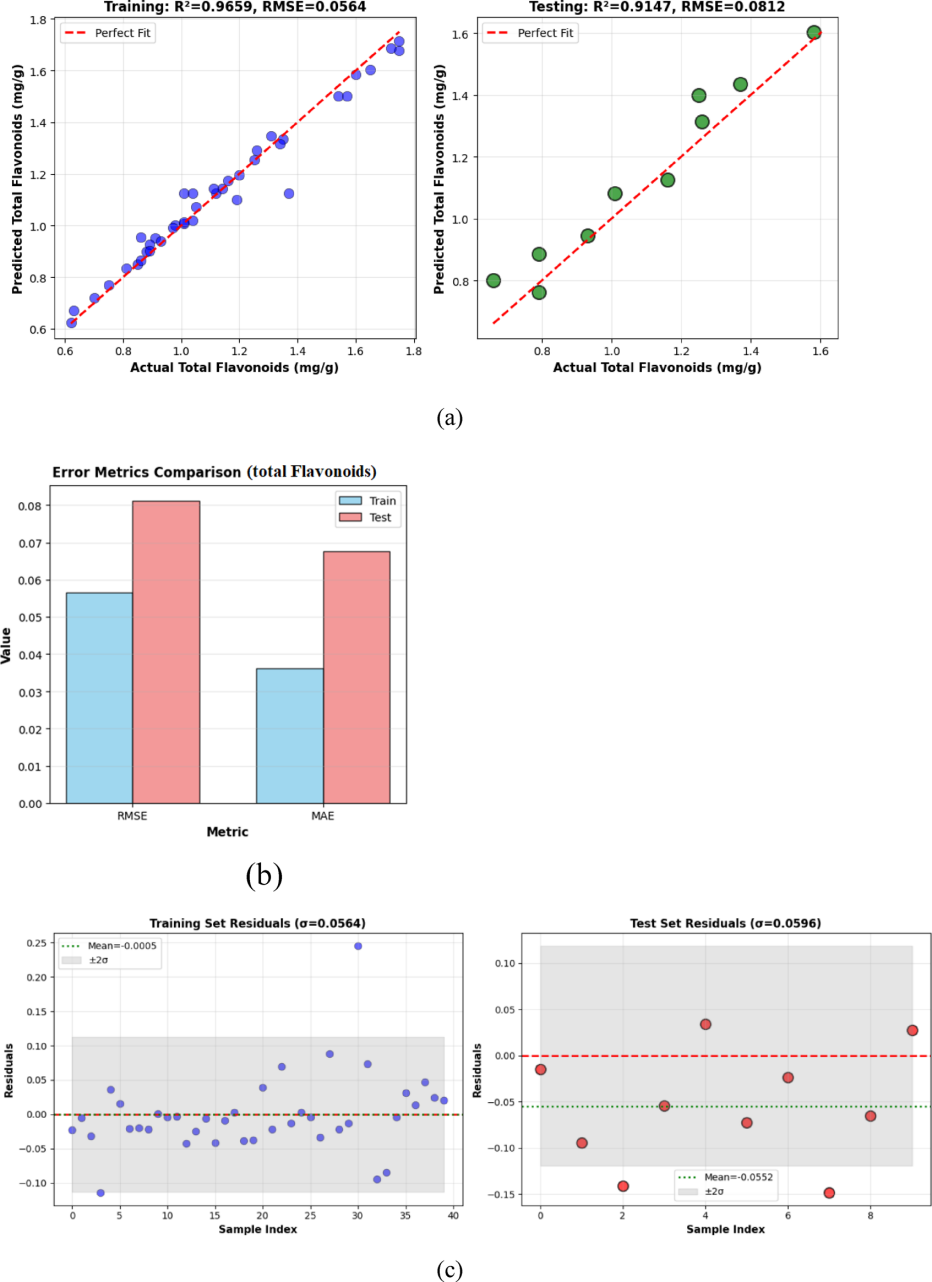
Performance evaluation of the Bayesian optimization-enhanced ensemble model for total flavonoid prediction. (**a** )Training and testing set prediction performance (**b**) Comparative analysis of error metrics (RMSE and mean absolute error). (**c**) Residual plots for training (left) and testing (right) sets.

The result for the Bayesian optimization-enhanced ensemble model’s performance is shown in Fig. 2. The model’s predictive capability for the training and testing data sets was outstanding, as shown in Fig. 2a. The training data set had an R² value of 0.9659 with an RMSE value of 0.0564 mg/g, while the testing data set had an R² value of 0.9147 and an RMSE value of 0.0812 mg/g. The difference in the values for the training and testing data sets, ΔR² = 0.0512, implies that there was little for overfitting, which means that the regularization techniques, including L2 leaf regularization, subsampling, and depth limitation, were successful in preventing the model from over complexification. The close fit of the predicted values and the perfect fit line for both the training and testing data sets implies that the model learned the relationships between the microwave extraction parameters and the flavonoid yield well. From the comparative error analysis shown in Fig. 2b, it is clear that there is a consistent performance of the model, as indicated by the mean absolute errors of 0.0373 mg/g and 0.0682 mg/g for training and testing sets, respectively. The ratios for the test set to train set for RMSE and mean absolute error ratios are 1.44 and 1.83, respectively, which are well within acceptable limits, indicating that there has been a successful trade-off between model complexity and its ability to generalize, as achieved by this ensemble approach. These values are very satisfactory, indicating a clear demonstration of the power of Bayesian optimization to find optimal hyperparameters for a model through systematic 5-fold cross-validation. The 5-fold cross-validation on the training set shows 0.8012 ± 0.0734. Residual analysis (Fig. 2c) offers a further validation of model quality. The training and testing data sets show a random distribution of residuals around zero (training data mean = −0.0005, σ = 0.0564; testing data mean = −0.0552, σ = 0.0596), with all data points lying within ± 2σ limits. The lack of systematic patterns, heteroscedasticity, or data points lying beyond ± 2σ limits ensures unbiased predictions across the complete range of the experimental design space (Microwave power: 100–500 W, Temperature: 40–70 °C, Time: 5–30 min, Mass/Volume Ratio: 20–40 mg/mL). The symmetric distribution of residuals around zero ensures model consistency and reliability, as it points to unbiased predictions, i.e., errors in predictions do not depend upon the magnitude of input parameters. Overall, these findings suggest that the model has the ability to achieve a high level of prediction accuracy (test R² > 0.91) and robust generalization capability, which makes the model appropriate for the optimization of the microwave-assisted flavonoids’ extraction process.

#### Total phenolic content (TPC) prediction

**Fig. 3:**
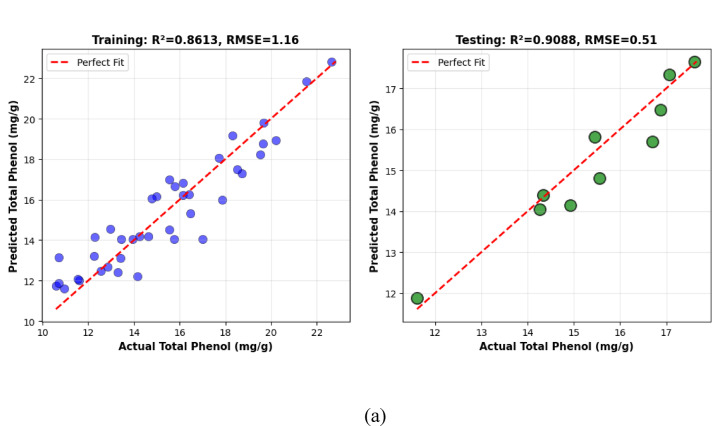

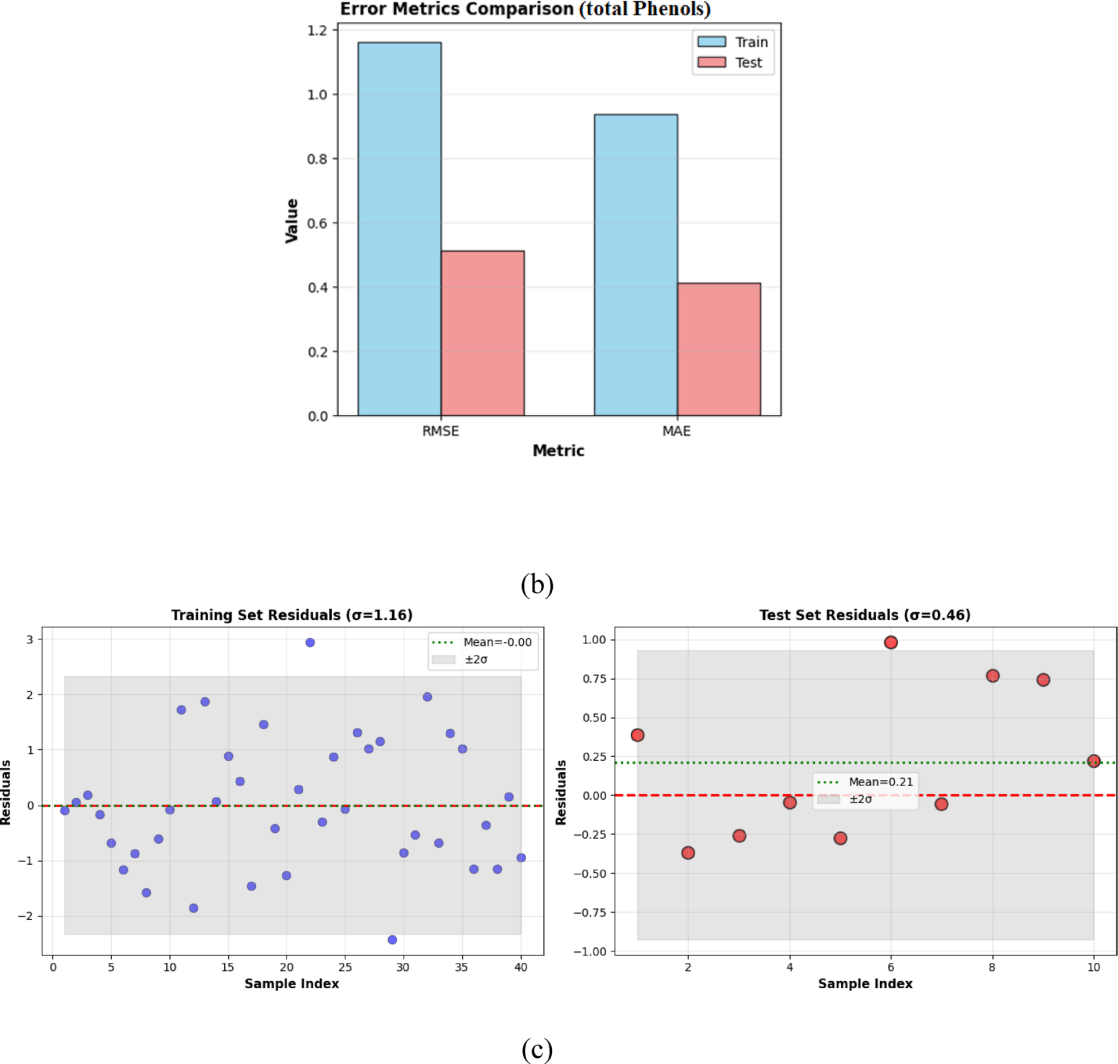
Performance evaluation of the Bayesian optimization-enhanced ensemble model for total phenol prediction. (**a**) Training and testing set prediction performance (**b**) Comparative analysis of error metrics (RMSE and mean absolute error). (**c**) Residual plots for training (left) and testing (right) sets.

The stacking ensemble model showed impressive predictive performance for total phenol content (TPC) across the entire range of the experimental design parameter space. As illustrated in Fig. 3a, the training data set yielded an R² of 0.8613 and an RMSE of 1.16 mg/g, whereas the test data set achieved even better performance with an R² of 0.9088 and an RMSE of 0.51 mg/g. It is worth noting that the relatively high R² value obtained from the test data set, compared to the training data set, is an unusual but favorable occurrence in the performance of ensemble models, wherein the performance of the meta-learner is able to generalize the diverse base model predictions without overfitting the training-specific noise. The predicted values closely follow the perfect fit line, with the test and training data sets showing good agreement, especially in the mid- to high-range values of the phenolic concentration (14–20 mg GAE/g). The comparative error analysis (Fig. 3b) shows that the testing errors were significantly lower compared to the training errors, considering both the RMSE and mean absolute error criteria. The results indicate that the model achieved good absolute accuracy in predictions, considering the phenol concentration range (10.59–22.65 mg GAE/g), as shown by the RMSE values: 1.16 mg/g (training) and 0.51 mg/g (testing). The lower test error, which corresponds to a very low RMSE ratio of 0.44 and mean absolute error ratio of 0.44, validates the effectiveness of the stacking model with proper regularization, which indicates that the complexity of the model was properly regularized to avoid both underfitting and overfitting. The choice to consider only the four original process parameters without additional feature engineering strategies turned out to be a strategic choice, considering that the inherent algorithmic diversity provided sufficient model capacity to capture non-linear relationships without increasing the risk of overfitting associated with a larger feature space, particularly considering the limitations of the available dataset. The cross-validation analysis (5-fold) showed consistent ensemble model performance of 0.8560 ± 0.0695, and independent testing results showed good accuracy with RMSE = 0.5706 mg/g and mean absolute error = 0.3442 mg/g. As shown in Fig. 3c, the residual analysis further confirms the quality of the developed model in terms of the lack of systematic bias. The training residuals showed a nearly zero mean (mean = 0.00), a standard deviation σ = 1.16, and a random distribution within the ± 2σ limits without any systematic patterns or heteroscedasticity. In addition, the test residuals showed a symmetric distribution around zero, with all the values falling well within the ± 2σ limits, thus validating the consistent quality of the predictions made by the developed model for unseen conditions. The lack of systematic patterns or trends in the residual plots for all the sample indices further confirms the independence of the prediction errors from the magnitude or sequence of the input parameters, thus validating the ability of the stacking ensemble model to effectively learn the extraction patterns from the experimental data. Overall, the above findings collectively confirm the high predictive accuracy and robust generalization ability of the developed Bayesian-optimized stacking model for the optimization of the microwave-assisted extraction process for the target compound.

#### DPPH radical scavenging activity prediction

**Fig. 4:**
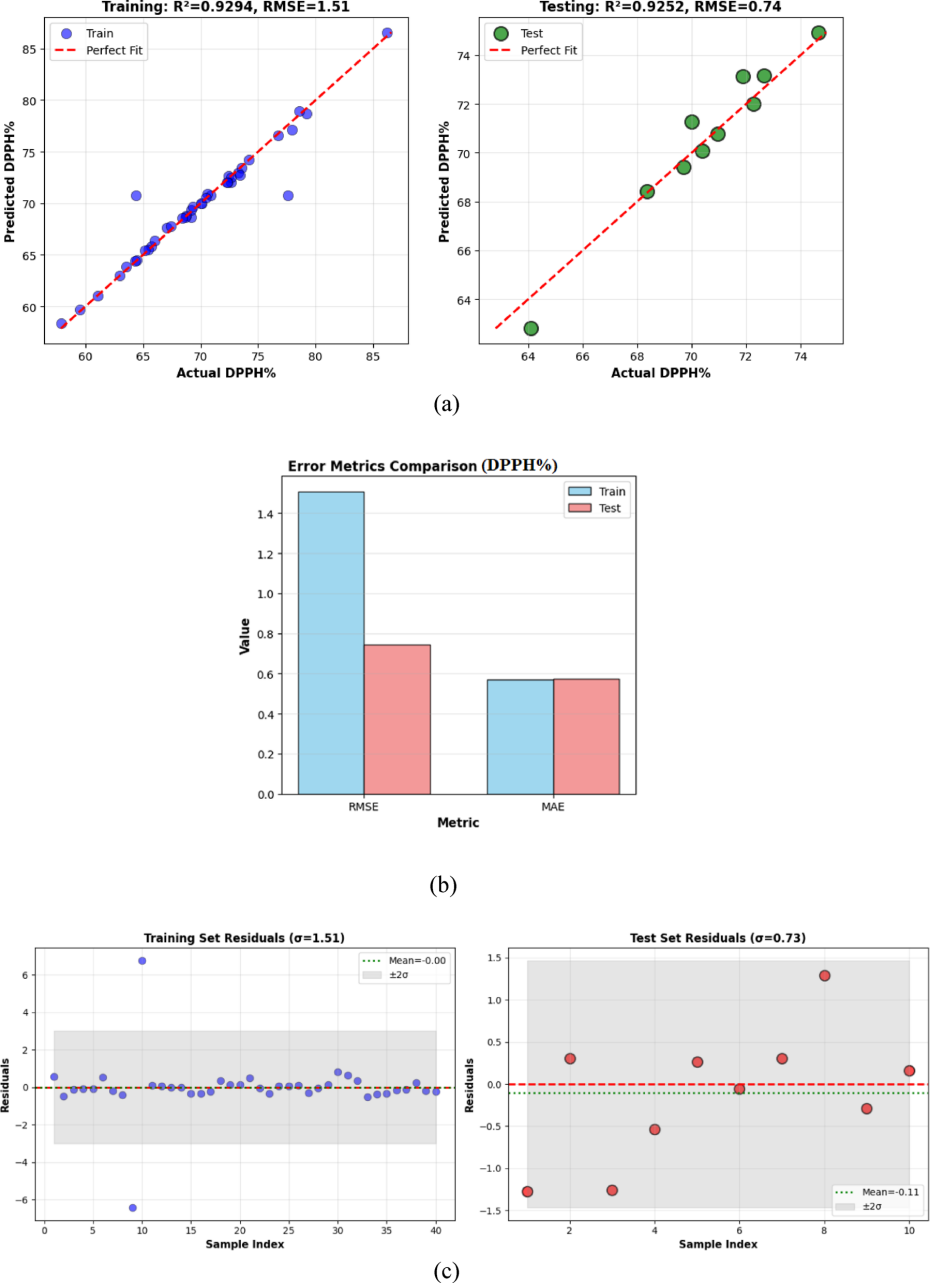
Performance evaluation of the Bayesian optimization-enhanced ensemble model for DPPH Radical Scavenging Activity (**a**) Training and testing set prediction performance (**b**) Comparative analysis of error metrics (RMSE and mean absolute error). (**c**) Residual plots for training (left) and testing (right) sets.

The ensemble testing and training data created by predicting DPPH activity resulted in outstanding performance of the ensemble model (Fig. 4a). The modeled training set has shown a highly predictable fit with an R²=0.9294 and RMSE = 1.51%, while the modeled testing set demonstrated a higher fitting accuracy than the fitting accuracy of the training set, R²=0.9252 and RMSE = 0.74%. The similarity of R² values (ΔR²=0.0042) of the two data sets facilitated and corroborated the generalization ability of the model and confirmed the effectiveness of the regularization techniques in addressing overfitting while providing a highly predictable model. The fitted values from the model also showed high correlations to the perfect fit line for the full range of DPPH% (58–87%) based on both training and testing data, most notably for mid/high antioxidant activity levels. As a comparative error metric (Fig. 4b) shows, the RMSE values of the training model (1.51%) and testing model (0.74%) resulted in high predictability. The decrease in the test RMSE compared to the training set RMSE (test RMSE is 0.49 times the training set RMSE) is a positive sign that confirms the ability of the model to generalize well and not memorize the training set data. The mean absolute error values of 0.58% for the training set and 0.58% for the test set show similar values for the average error in the predictions made by the model. This confirms the ability of the model to generalize well for both the training set and the test set. This shows that the typical error in the predictions made by the model is less than 1% DPPH inhibition activity, which is highly acceptable due to the experimental error associated with the DPPH assay. This error profile confirms that the Bayesian optimization process was successful in identifying the hyperparameters that maximize the accuracy of the predictions made by the model. In other words, the 5-fold cross-validation results revealed that the model had a high predictive power of 0.9201 ± 0.1380, although there was some variation due to the small size of the dataset, which was further confirmed by the independent test set results. The residuals plot (Fig. 4c) further offers confirmatory evidence of model quality and the lack of systematic prediction bias. The training residuals had a near-zero mean value (mean = −0.00) with a standard deviation of σ = 1.51. The random distribution of residuals within the ± 2σ range for all index values further offers confirmatory evidence that prediction bias was independent of DPPH% value or experimental sequence. The testing residuals had a similar distribution pattern with a mean value of −0.11 and a standard deviation of σ = 0.73. All values were well within the ± 2σ range, further confirming the model’s ability to generalize well to unseen data. The lack of systematic trends in the residuals plot further offers confirmatory evidence that the ensemble model successfully learned generalizable patterns governing antioxidant activity rather than fitting to dataset-specific bias. The findings confirm that the optimized ensemble model offers high predictive accuracy with good generalization ability, thus being useful in the optimization of the microwave procedure for enhanced antioxidant capacity.

### Feature importance

**Fig. 5:**
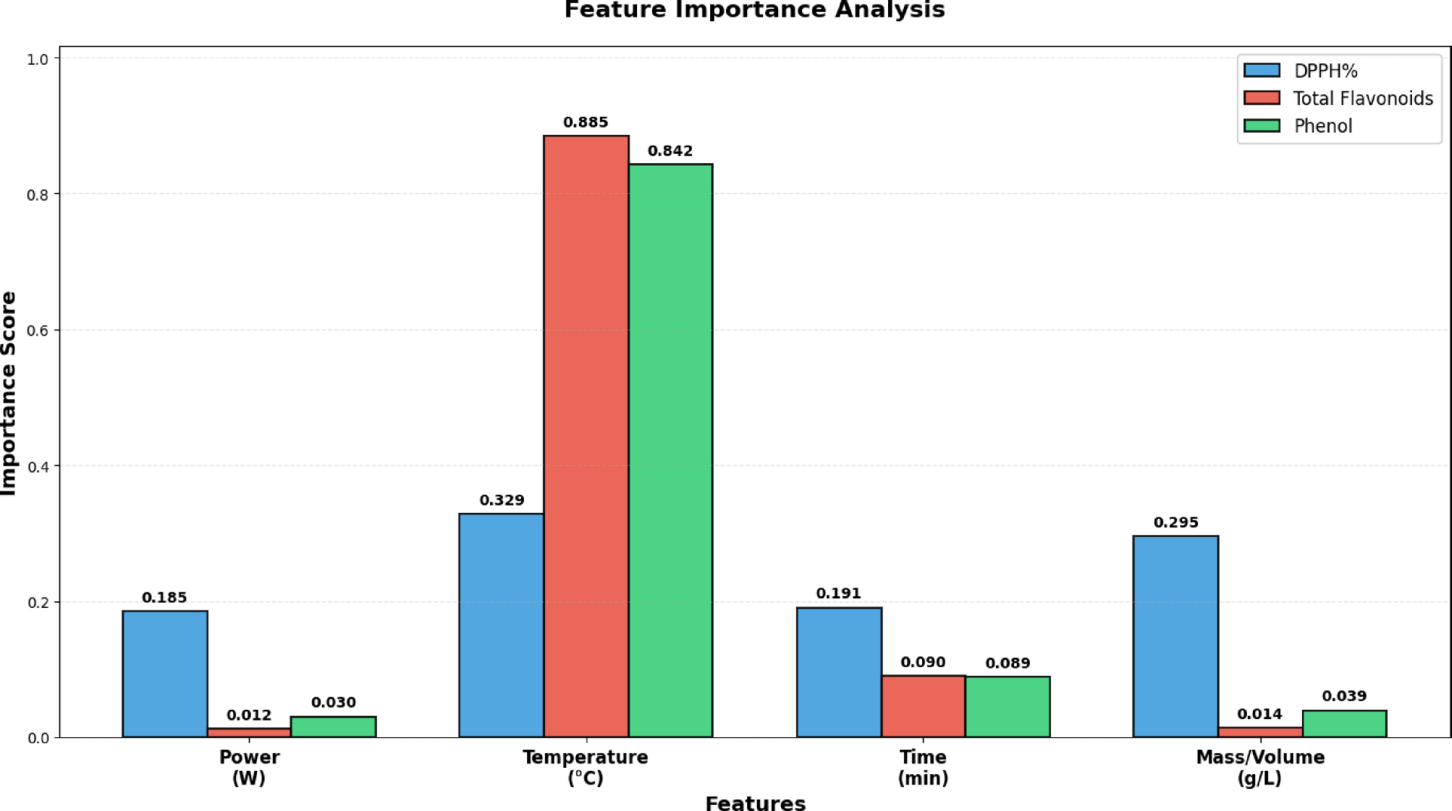
Feature importance analysis of extraction parameters on bioactive compound yields and antioxidant activity.

Feature Importance Analysis shone a light on the various extraction parameters and how much they impact the yield of bioactive compounds and their antioxidant qualities. The results demonstrated that temperature had the largest effect on all response variables. The order of importance was total flavonoids (0.885), total phenols (0.842), and DPPH% (0.329). High temperature is directly correlated to how effectively the heat will destroy plant cell walls and release phenolic compounds. The second most important factor affecting the response variables was the mass/volume ratio, which was most important in the determination of DPPH% (0.295). The factor was less important in the determination of flavonoids (0.014) and total phenols (0.039). The power and time factors were less important in the determination of all response variables, with power being slightly more important in the determination of DPPH% (0.185) than in the determination of total phenol (0.012, 0.030). Temperature plays the most dominant part (importance 0.842–0.885) due to several reasons: higher temperature raises the molecular kinetic energy and increases diffusivity, lowers the solvent viscosity, which facilitates the solvent movement in the cell structure, causes hydrogen bond disruption of phenolic compounds with cellulose in the plant matrix, and increases the mass transfer at the solid-liquid boundary^16,18^. Solid-to-liquid ratio regulates equilibrium and concentration gradient conditions during extraction; a higher solid-to-liquid ratio means the solvent occupies a smaller volume, creating unfavorable diffusion conditions; it is especially important in the case of diffusion-controlled extraction of flavonoids. Microwave power affects the energy deposition rate; in case of too much power used during the process, overheating may occur, leading to flavonoid thermal degradation, therefore being less important compared to the temperature factor. Time affects the possibility of mass transfer through the cell structure by providing more time for extraction; however, excessive time results in flavonoid oxidation, and therefore, there is an optimal time interval that can be proven during the multi-response optimization (Figure 5).

**Fig. 6:**
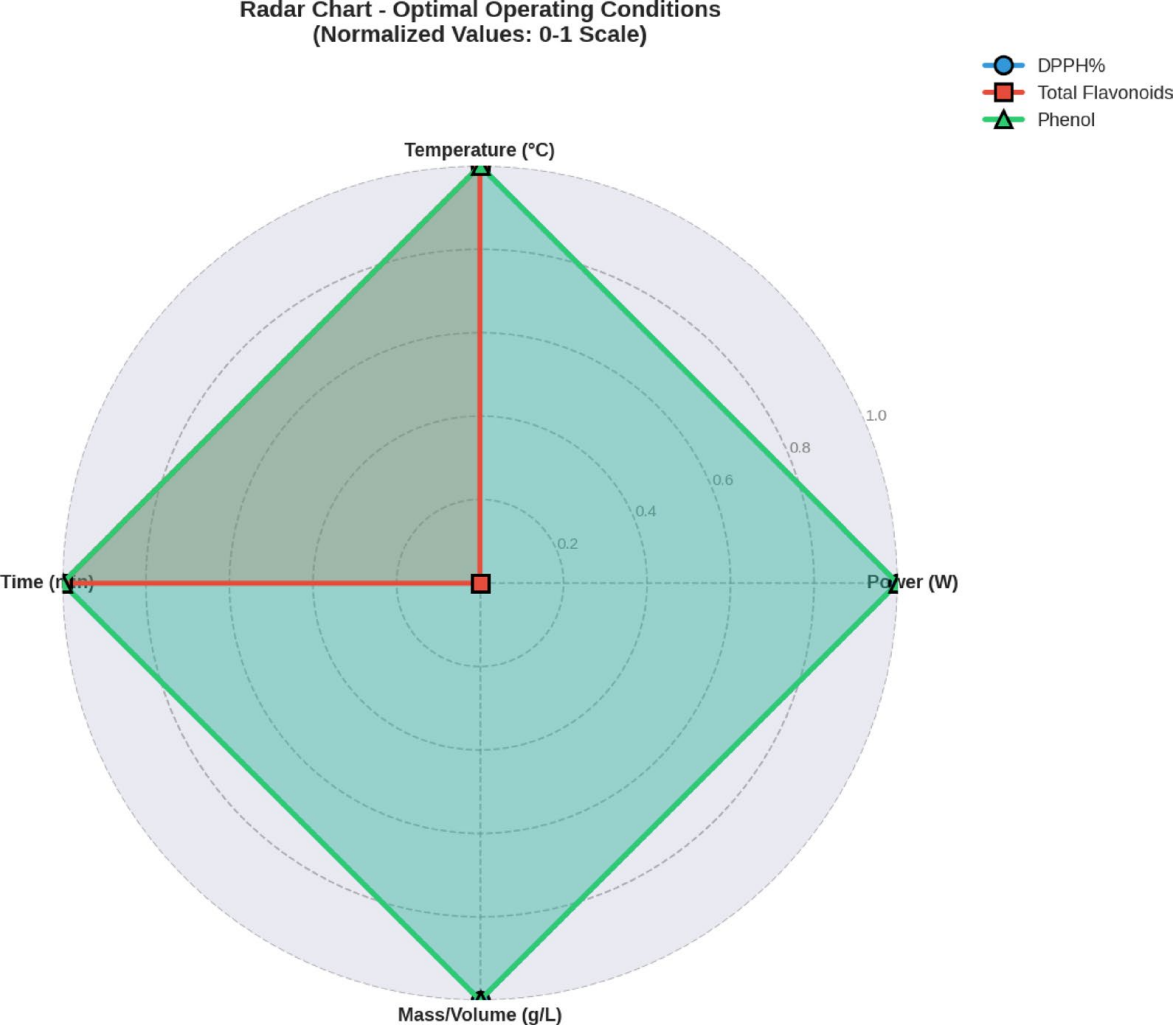
Radar chart visualization of normalized optimal operating conditions across extraction parameters for different bioactive responses.

To better illustrate the differential sensitivity of response variables to extraction parameters, a radar chart was plotted using normalized optimal operating conditions (Fig. 6). The results showed that each of the three response variables has its own pattern of responses. For total phenol, it was found to be highly sensitive to all four parameters. The normalized values of total phenol approached the highest value of 1.0 for all parameters, indicating that total phenol extraction is highly dependent on the optimization of several parameters. For total flavonoids, it was found that this compound is highly dependent on time and temperature but is less sensitive to power. The normalized values of total flavonoids approached the highest value of 1.0 for time and temperature but remained low for power. For DPPH%, it was found that this compound is moderately dependent on all four parameters. The normalized values of DPPH% ranged from 0.3 to 0.6 for all parameters. It appears that this compound is dependent on the combined effect of several parameters. The diamond profile of phenol, in contrast to the asymmetric responses of flavonoids and DPPH%, indicates the complexity of microwave-assisted extraction, which shows that different bioactive compounds react in different ways to the same extraction conditions, thus requiring a multi-response optimization strategy to attain the maximum efficiency of the extraction process.

### Multi-response process optimization

**Fig. 7:**
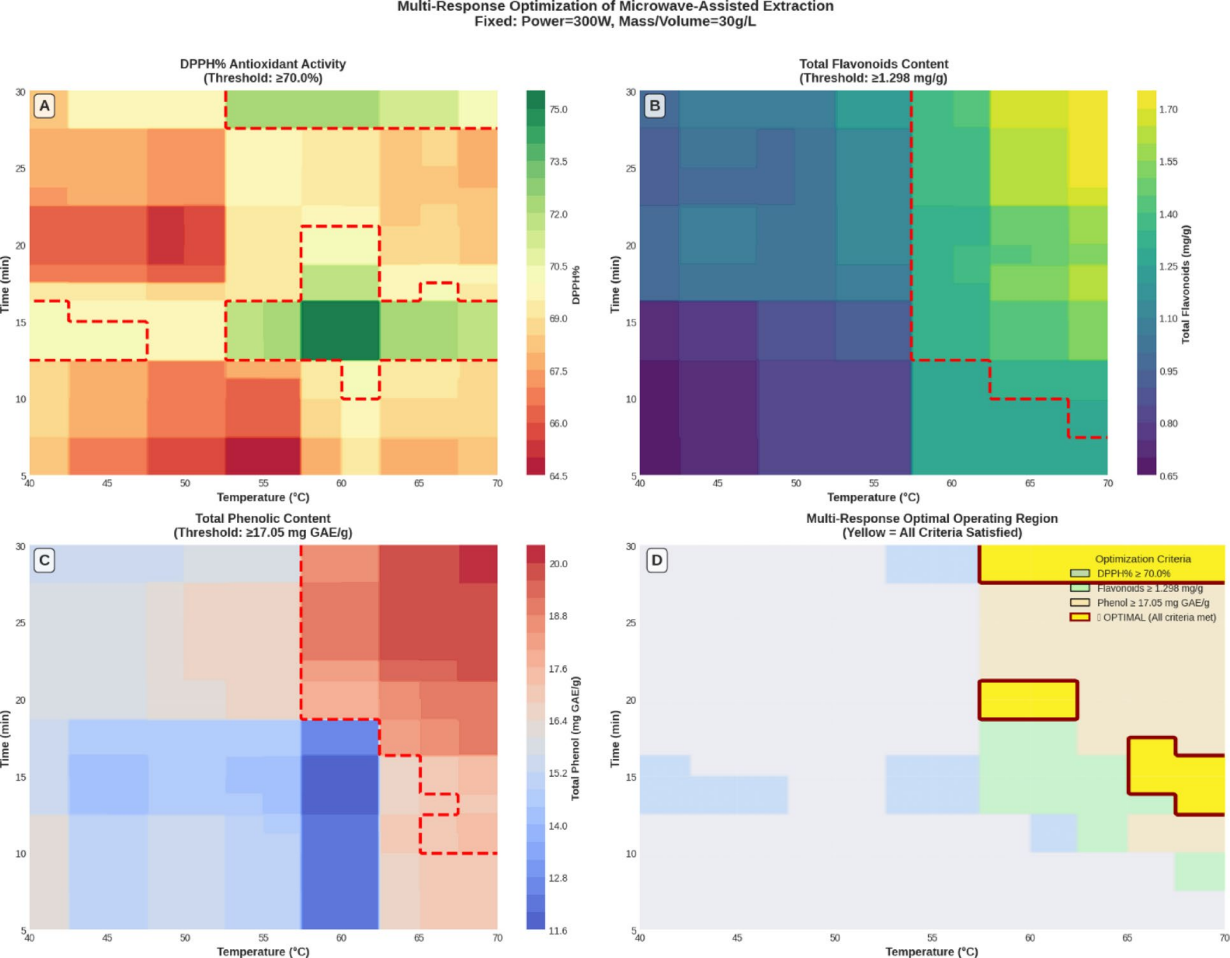
Process optimization maps showing individual optimal regions for DPPH activity (top left), total flavonoids (top right), and total phenols (bottom left) as functions of temperature and time. The multi-response overlay (bottom right) identifies the common optimal operating region (yellow with red border).

Ensemble machine learning models were used to determine the optimum conditions that meet the various quality criteria simultaneously (Fig. 7). Response surface heatmaps were produced with respect to temperature (40–70 °C) and time (5–30 min) with fixed power (300 W) and solvent/material ratio (30 mg/mL). These constant values represent the optimal levels generated by the model based on the multiple response optimization approach and coincide with the centroid of the optimal region; they have not been chosen arbitrarily. The heatmaps demonstrate the existence of separate optimum conditions for every response (Fig. 7a-c). DPPH% had a wide range of optimum conditions (57–70 °C, 12–30 min), indicating strong antioxidant activity over a wide range of conditions. Total flavonoids had high optimum conditions with respect to both temperature and time (60–70 °C, 20–30 min), corresponding to the increased cell wall disruption requirements. Total phenolic content had intermediate conditions (58–70 °C, 15–30 min). The multi-response overlay plot (Fig. 7d) revealed a common optimal area (8.37% design space) where all three quality characteristics were simultaneously satisfied. The optimal point was found to be 62.5 °C and 27.7 min, which predicted DPPH% to be 71.27%, total flavonoids to be 1.656 mg/g, and total phenol to be 19.80 mg GAE/g, employing the desirability function optimization. The accuracy of the model was validated by carrying out experiments in triplicate, and it was found that the model had excellent accuracy with a relative error of 2.12%, 1.87%, and 2.53% for DPPH%, flavonoids, and phenols, respectively (Table 3). All values were found to be higher than the predicted values, which ensured that the optimization process would be conservative and applicable in industry.

For the validation of the predictive precision of the multi-response optimization approach, experimental verification was performed on the established optimal point of operation (temperature: 62.5 °C, time: 27.7 min, power: 300 W, mass/volume: 30 mg/mL), which coincided with the centroid of the unified optimal region (Fig. 7, yellow overlay). Table 3 displays the comparison of the model predictions and the experimental measurements for triplicate extractions.

**Table 3:** Experimental validation of optimal extraction conditions.

Process parameters (optimal point)					
Temperature			62.5 °C		
Extraction time			27.7 min		
Microwave power			300 W		
Mass/volume ratio			30 mg/mL		

Bioactive response performance					
Response	Predicted value	Experimental value	Absolute error	Relative error (%)	
DPPH activity (%)	71.27	72.78 ± 1.24	1.51	2.12	
Total flavonoids (mg/g)	1.656	1.687 ± 0.034	0.031	1.87	
Total phenols (mg GAE/g)	19.8	20.3 ± 0.41	0.5	2.53	

Experimental values represent mean ± standard deviation from triplicate extractions.

Excellent agreement has been found between the predicted and experimental data, with relative errors of 2.12%, 1.87%, and 2.53% for DPPH%, TFC, and TPC, respectively. This level of error is well within the acceptable limits for the optimization of the process using the ensemble-based approach, thus validating the training of the model and the proposed multi-response optimization strategy. As the experimental validation of the model has been conducted at the optimum condition of 60–65 °C and 20–23 min, the simultaneous attainment of the three objectives has been demonstrated, thus validating the proposed optimization approach for the process. As the prediction errors of < 3% have been achieved for the entire response surface, it has been demonstrated that the proposed models can be used to select the extraction parameters without the need for trial and error.

The coupling of machine learning-based optimization with experimental verification is an effective strategy for designing efficient and sustainable extraction methods. The Bayesian optimization approach was able to effectively explore the complex, nonlinear parameter space with a surprisingly high degree of computational efficiency, requiring only 30–50 iterations to reach convergence, as opposed to the very high number of experiments required by traditional methodology. The ensemble learning model architecture, which combines multiple base models with different algorithmic strengths, was shown to have superior predictive accuracy and robustness compared to individual models, successfully modeling both linear and complex interaction effects among extraction parameters. The small prediction errors obtained during experimental verification (< 3% relative error) validate the practical accuracy of the developed models for process optimization. Additionally, the feature importance analysis provided a mechanistic understanding of the process, indicating that temperature is the dominant factor in the extraction process due to its ability to facilitate cell wall rupture and improve mass transfer, while the solid-liquid ratio affects the extraction equilibrium and solvent accessibility. The successful identification and validation of optimal conditions that meet multiple quality criteria demonstrate the effectiveness of the computational framework for multi-objective optimization in food bioprocessing applications. The results above collectively form a data-driven methodology that can be extended to other agricultural waste valorization processes, thus contributing to the goals of circular bioeconomy and sustainable food systems development.

A comparison of the obtained results was conducted with published results on the extraction of watermelon rind compounds. The study conducted by Zia et al.^4^, for instance, used ultrasound-assisted extraction and reported TPC in the range of 1–2.5 mg/g based on various conditions of the experiment. The same applies to another study, where TPC was reported as 28.55 mg GAE/g^26^ and 0.026 mg GAE/g^27^. Differences among reported values may be affected by extraction technique, solvent system, raw material characteristics, and experimental conditions. Under the optimized conditions of the present study, the TPC of 19.80 mg GAE/g, 1.656 mg CE/g (TFC), and 71.27% DPPH activity was obtained. The obtained extraction values are comparable to, and in some cases higher than, several literature-reported studies on watermelon rind extraction; however, direct experimental comparison was beyond the scope of the present study. This enhanced extraction efficiency can be attributed to the synergistic effects of fermentation pretreatment, NADES as a green solvent system, and microwave-assisted extraction,

which collectively improve cell wall disruption, solvent penetration, and mass transfer of bioactive compounds. From an extraction yield perspective, in the case of optimum extraction condition (solid/liquid ratio 30 mg/mL, TPC = 19.80 mg GAE/g dry weight), one ml of NADES extract can be obtained using approximately 0.594 mg GAE total phenolics, showing high extraction yield efficiency per unit volume of solvent. Total extractable content determination as part of a complete mass balance is highly suggested as a future research direction. Furthermore, to ensure distinction between the effects of the fermentation process and the extraction process, it must be noted that during the fermentation comparison analysis, the same fixed conditions were maintained in the extraction phase (50 °C, 100 W, 20 min, 20 mg/mL) to focus on the fermentation process alone.

## Conclusion

This research achieved the optimization of the bioactive extraction from watermelon rind through the combination of natural deep eutectic solvents (NADES), microwave-assisted extraction (MAE) and solid-state fermentation methods using advanced machine learning models. Watermelon rind is a large volume of agricultural waste globally. The fermentation process greatly improved the extractability of phenolic and flavonoid compounds, leading to 18–34% increases in bioactive compound yields over non-fermented controls, validating the effectiveness of enzymatic cell wall degradation in improving downstream extraction efficiency. The Bayesian optimization-assisted ensemble machine learning model developed in this work attained outstanding predictive performance (R² > 0.91) with strong generalization ability (ΔR² < 0.06) for all three response variables, illustrating the effectiveness of computational intelligence in modeling complex bioprocesses. Importance analysis of the feature variables revealed temperature (40–70 °C) as the most important extraction factor, with importance coefficients above 0.84 for phenolic and flavonoid extraction, offering essential mechanistic information for process optimization. The multi-response optimization problem was successfully solved to identify a single optimal region (62.5 °C, 27.7 min, 300 W, 30 mg/mL) that simultaneously maximized total phenolic content (19.80 mg GAE/g), total flavonoid content (1.656 mg CE/g), and antioxidant activity (71.27% DPPH inhibition), and the results were experimentally validated with 3% relative error. The computational framework developed in this study was able to achieve a reduction of about 75% in the number of experiments required compared to the traditional method, while still producing optimal results. The bioactive compounds isolated in this study have a high level of antioxidant activity, which makes them useful for the development of nutraceuticals and pharmaceuticals.

## Data Availability

The experimental data and machine learning models generated during this study are available from the corresponding author upon reasonable request.
